# Commentary to “Lower middle cerebral artery blood velocity during low-volume high-intensity interval exercise in chronic stroke”

**DOI:** 10.1177/0271678X241235885

**Published:** 2024-02-28

**Authors:** Andrew D Robertson

**Affiliations:** 1 Schlegel-UW Research Institute for Aging, University of Waterloo, Waterloo, ON, Canada; 2 Department of Kinesiology and Health Sciences, University of Waterloo, Waterloo, ON, Canada

High-intensity interval training has emerged as a promising tool for stroke rehabilitation.^1^ Implementation of high-intensity interval paradigms into clinical rehabilitation permits individuals to optimise the metabolic, cardiopulmonary, and functional benefits of exercise^2,3^ while limiting duration and circumventing the barriers imposed by post-stroke fatigue.^4^ Safety concerns linked to periodic high cerebral perfusion pressure during high-intensity interval exercise (HIIE) in persons with stroke have been tempered by feasibility assessments,^5^ as well as by more recent adverse event reporting following a randomised clinical trial.^3^ The overwhelming majority of research in this field, however, has failed to assess cerebrovascular responses during such an acute stimulus in individuals who may have residual compromised cerebrovascular health.^6^ In this issue, Whitaker and colleagues report on the cerebrovascular response during a single bout of HIIE in individuals with chronic stroke (n = 25) compared to age-matched controls (n = 25).^7^ This represents an important step in furthering our understanding of the role of HIIE in stroke rehabilitation, but also an appropriate point of reflection as to how we move forward.

## Physiological underpinning

The HIIE protocol used in this study involved 1-minute intervals, alternating between 70% and 10% estimated maximum power, for 10 minutes. The authors showed lower ipsilesional middle cerebral artery blood velocity (MCAv) at rest in persons with chronic stroke, which persisted during, and for up to 30 minutes after, exercise. MCAv was lower in persons with stroke during both high intensity and active recovery phases of HIIE. Interestingly, group-wise relative increases in MCAv peaked at ∼17% in persons with stroke, which is comparable to changes reported during moderate-intensity continuous exercise (MICE).^8^ Posited mechanisms related to cerebrovascular benefit of HIIE include exposure to increased mechanical stressors (e.g., shear rate),^2^ yet the current findings would suggest minimal differences between MICE and HIIE. Of further interest is a concept introduced by Whitaker and colleagues as MCAv responsiveness – the variability of the fluctuations in MCAv between the high intensity and active recovery intervals. While this “responsiveness” was lower in persons with stroke, the fluctuations in MCAv – and thus shear stimulus – may yet prove to be a beneficial aspect of HIIE for cerebrovascular health. Responsiveness in this context might be better examined with greater temporal resolution to characterize peak MCAv observed during transitions between intervals rather than simply averaged across the interval.

## Statistical clarity

Characterizing the response to acute exercise is critical to further our understanding of the physiological mechanisms underlying potential gains in cerebrovascular health following training interventions. Importantly, appropriate statistical approaches are essential to provide clear understanding and interpretation of these responses. While Whitaker and colleagues aimed to characterize differences in mean MCAv responses between persons with chronic stroke and control participants, the primary linear model was lacking both baseline values and a group-by-time interaction term. Indeed, the sensitivity analysis that included baseline values eliminated overall group differences, thereby emphasizing the point that baseline values are essential to primary statistical models designed to assess between-group responses when baseline group differences exist. Furthermore, without a statistical interaction term, we are unable to properly infer whether the response in persons with stroke was different than controls, but instead are restricted to inference based on the main effects of group and time.

## Clinical importance

In view of the heterogeneous pathophysiology of stroke, it is notable that one quarter of this study’s clinical cohort had a hemorrhagic etiology (n = 6). While few in number, that absence of any adverse events adds to a growing evidence base that HIIE is safe and feasible for persons with chronic stroke, regardless of type.^3,5,9^ Still, a lack of trials currently dissuades clinical recommendations from endorsing HIIE prescription for stroke rehabilitation.^10^ Another issue delaying more widespread clinical implementation is the lack of consensus on appropriate duty cycles of the high-intensity and active recovery exercise. The 1-minute 1:1 cycle used by Whitaker and colleagues has been shown to improve gait function, whereas longer cycles (e.g., 4 minutes on/4 minutes off) induce greater lactic anaerobic stimuli that may convey greater metabolic and cardiorespiratory benefits. Given demonstrated safety and efficacy, multi-modal rehabilitation programs imparting both short- and long-duration intervals may be advantageous.^3^

## Future directions

As we continue the search for optimal rehabilitative strategies to meet the ever-widening needs of a diverse population living with stroke, high-intensity exercise modalities appear to be important tools. Understanding the nuances of cerebral blood flow dynamics within transitions during HIIE, as well as in response to different HIIE paradigms, is still needed. Furthermore, following the demonstration of feasibility and safety of HIIE in persons with chronic stroke, we may begin to ask the questions: At what stage post-stroke is risk tolerance acceptable for HIIE? Is posterior cerebral blood flow more sensitive to physiological fluctuations induced by HIIE? What is the role of persistent hemodynamic and metabolic factors, such as the saw-tooth pattern observed in blood pressure and end-tidal CO_2_? For now, this work by Whitaker and colleagues provides an important step to understanding the role of cerebral hemodynamic responses to exercise in the benefits of longer-term rehabilitation strategies.
